# Pressure injury: update on general concepts, clinical aspects, and laboratory findings – Part I^؆^

**DOI:** 10.1016/j.abd.2025.501187

**Published:** 2025-08-18

**Authors:** Bruna Cristina Velozo, Michelle Venâncio Hong, Larissa Cassiano Bernardo, Meire Cristina Novelli e Castro, Jose Contreras-Ruiz, Luciana Patricia Fernandes Abbade

**Affiliations:** a Department of Nursing, Faculty of Medicine, Universidade Estadual Paulista, Botucatu, SP, Brazil; b Polanco Dermatological Center Los Cabos, Dr. Manuel Gea González General Hospital, Mexican Institute for Advanced Wound Care, Mexico; c Department of Infectology, Dermatology, Imaging Diagnosis and Radiotherapy, Faculty of Medicine, Universidade Estadual Paulista, Botucatu, SP, Brazil

**Keywords:** Classification, Epidemiology, Pressure ulcer, Risk factors

## Abstract

Pressure injuries remain a significant challenge in healthcare services. They negatively impact these services by increasing workload, financial burden, and direct and indirect costs related to detection, prevention, treatment, and rehabilitation. It is of utmost importance that dermatologists and other healthcare professionals are knowledgeable about these lesions. The first part of this review discusses the history and terminology of pressure injuries, epidemiology in different settings, from adults to pediatric and neonatal patients; etiopathogenesis, demonstrating the current scheme of the vicious cycle of ischemia and tissue death; associated risk factors, both intrinsic and extrinsic; classification of all stages of pressure injuries with clinical images; and the main anatomical areas at risk in each position ‒ lateral, seated, supine, prone, and with medical devices. Differential diagnoses were detailed, including incontinence-associated dermatitis, and the “Kennedy terminal ulcer”, providing support for proper evaluation and guidance on preventive measures and treatment, which will be further detailed in Part II of this review. The primary focus was to provide resources for healthcare professionals to holistically assess, prescribe, and monitor patients at risk for or with pressure injuries.

## Introduction

The pressure injury (PI), also known as a “pressure ulcer”, is a localized area of injury to the skin and/or underlying tissue, usually over a bony prominence. It may present as an area of seemingly intact skin with non-blanchable erythema, blistering, or an ulcer with complete loss of skin and possible involvement of underlying deep tissues such as muscles and bones. Medical-device-related PI was defined as resulting from the use of devices designed and applied for diagnostic or therapeutic purposes. The resulting PI conforms to the device’s pattern or shape.^1^

PI is a persistent healthcare condition. It represents one of the most challenging clinical problems that negatively impact patients' lives in emotional, psychological, physical, and social aspects, thus globally reducing their quality of life.^2^ PI also has repercussions on healthcare institutions, leading to increased workload and financial burden. Unfortunately, its incidence and burden remain at high levels,^3^ mainly after the COVID-19 pandemic.^4^

The economic impact of PI includes expenses for detection, prevention, treatment, and rehabilitation.^5^ The cost of treating a PI per patient per day varies between 1.71 and 470.49 euros in all scenarios.^6^ In Intensive Care Units (ICUs), PI can generate even higher costs, especially with more severe and deeper wounds.^5^

Based on the best available scientific evidence, this review highlights the importance of dermatologists and other healthcare professionals knowing about Pressure Injuries (PIs), particularly their ability to diagnose and differentiate PIs from other similar lesions effectively. Additionally, it considers the most current mechanisms of PI development, classifications, and clinical aspects to support healthcare professionals in their clinical routines. This includes assessing patients for potential risk factors to take assertive preventive measures and facilitating early identification of PIs to intervene and prevent complications promptly.

### History of pressure injuries

PIs have been a part of human history since ancient times. The earliest known historical reference comes from paleopathologists who identified extensive PI on the ischial bones and scapulas of a mummy belonging to an elderly priestess of Amun from the 21^st^ Dynasty of ancient Egypt (1070 to 945 BC).^7^ The first written record of PI corresponds to Hippocrates (460‒370 BC), who described the appearance of such lesions in a paralyzed patient with bladder and bowel dysfunctions.^8^

During the Renaissance, Ambroise Paré (1510‒1590), considered the father of modern surgery, made the first documented description of a PI, focusing on addressing its cause to facilitate healing.^7^

Effective measures in the prevention and treatment of PI were already being implemented in the early 19th century. For example, in 1806, J.E. Aronssen from Berlin emphasized the importance of the bed in patient care and presented plans for a new and improved invalid chair of his invention.^9^

The term “bedsore” was documented by nurse Florence Nightingale in 1859.^10^ This term maintains its association with the bed, despite the understanding at the time that PI can occur whenever soft tissues are in contact with support surfaces, and the significant role played by shear forces and shear deformation. The addition of “sore, implied a raw” or painful place on the body.^1^

From the second half of the last century, awareness of PI increased alongside advances in healthcare, prompting various authors to study their etiology. Reichel in 1958^11^ and Kosiak in 1959^12^ with studies on ischemic injuries contributed significantly to the understanding of etiopathogenesis. The search for Norton et al. in 1962^13^ resulted in the first risk assessment scale for PI. The term “pressure ulcer” started to gain popularity in the 1970s and 1980s, replacing terms like “bedsores” or “decubitus ulcers” and “pressure sores”. The formation of groups such as the National Pressure Ulcer Advisory Panel (NPUAP) in the United States in the 1970s, along with national groups such as the National Group for the Study and Advisory of Pressure Ulcers and Chronic Wounds in Spain (GNEAUPP) in 1994, and the European Pressure Ulcer Advisory Panel (EPUAP) in 1996, propelled the advancement of knowledge about PI during that time.^7^

Significant contributions to the understanding and prevention of PI include the conceptual model by Bergstrom et al. in 1987^14^ who developed the Braden Scale, and Coleman et al. who presented direct and indirect causal factors,^15^ and the work of García Fernández et al. in 2014,^16^ who introduced the term “pressure injuries related to dependency”. This work modified the existing classification that GNEAUPP had already adopted and paved the way for reconsidering the term “pressure injuries” instead of “pressure ulcers”. This term was later adopted by the Pan Pacific Pressure Injury Alliance in 2014 and recently, albeit controversially, by the American NPUAP, generating intense debate due to the English connotations of the concept of “injury”.^17^ Thus, the NPUAP incorporated the “injury” (National Pressure Injury Advisory Panel ‒ NPIAP),^1^ however, in Europe, the EPUAP term pressure ulcer remains without this modification.^1^

## Epidemiology

Assessing PI incidence is of utmost importance in demonstrating the relevance of this adverse event in hospital settings. It is crucial for healthcare professionals at the bedside and service managers, as it indicates the quality of care provided,^18^ becoming a threat to patient safety.^19^

The prevalence of PI remains high in the global population, proportional to aging and the increasing prevalence of precipitating factors such as obesity, diabetes, and cardiovascular diseases.^20^ Conversely, the incidence varies widely according to the clinical setting and is higher among high-risk groups. It is reported to be up to 60% among quadriplegics and people with spinal cord injuries, 66% among patients undergoing prolonged surgeries, and 70% among older people patients with hip or femoral fractures.^20^

The global prevalence of PI reported in a systematic review in hospitalized patients is 12.8%, with an incidence rate of 5.4 per 10,000 patient days.^21^ The same review found that 61.8% of PIs were acquired in hospitals, and 30% of all lesions were considered severe (Stages 3 and 4).^21^

Brazil is the country that reports the most studies on the incidence and prevalence of PI in Latin America.^22^ According to the National Report on Healthcare-Related Incidents reported to the National Health Surveillance Service from 2014 to 2022, out of 1,100,352 reported incidents, 223,378 (20.3%) were related to PIs. During this period, PIs were the second most frequently reported type of event by the Patient Safety Nucleus (NSP) of healthcare services in the country and the fifth leading cause of death related to incidents in healthcare.^23^

Data on the prevalence and incidence of PI should be analyzed cautiously in different studies since authors use different methodologies to calculate these indicators. The main problems are the inclusion of all stages of PI or only those with ulcers and the inclusion of the entire population or only those at risk of developing PI. These differences significantly impact the incidence and prevalence results found in different studies and countries. This limitation has already been found in a systematic review, for example, where not all stages of PIs were included in the studies.^21^

### Intensive therapy

In ICUs, patients are considered at high risk for developing PI due to clinical condition-related risk factors, such as immobility, inadequate nutrition, and support therapies like mechanical ventilation and hemodialysis.^19^, ^24^ The cumulative incidence and prevalence were 10.0% to 25.9% and 16.9% to 23.8%, respectively in a global systematic review encompassing all diagnostic restrictions and types of ICU.^24^

### Pediatric and neonatal

The prevalence and incidence of PI in pediatric populations can vary considerably depending on the clinical context. Few studies focus on this population, but Razmus and Bergquist-Beringer conducted a study covering 678 pediatric acute care units with 39,984 patients in the United States.^25^ They reported that the rate of PI acquired in all facilities ranged from 0.57% to 3.7% in the pediatric ICU and 4.6% in rehabilitation settings.^25^

A study in Brazilian hospitals, tracking 314 children, found an average prevalence of 7.1% and an average incidence of 21.8%, with 22 children having 31 cases of PIs. According to the types of wards, the average prevalence was 1.8% in clinical pediatrics, 4.0% in surgical pediatrics, and 32.8% in pediatric ICUs.^26^ In another Brazilian study assessing 83 children in a tertiary hospital ICU in the interior of São Paulo, the incidence of PI was 21.6%.^27^

In neonates, injuries from medical devices are more common, especially in the ICU.^28^ A study that assessed the incidence of PI in both children and neonates identified a 50% higher risk of PI, primarily due to medical devices, with injuries to the septum from enteral tubes and the use of tracheostomies.^29^ Another study evaluated 625 children and neonates, among which those under eight years old developed one or more injuries from medical devices, with an incidence of 7%.^30^

A recent systematic review found the overall pediatric prevalence ranging from 0.47% to 31.2%. The combined prevalence among neonates was 27.0%, among children under 1-year old was 19.2%, and among children over 1-year old was 12.3%. The cumulative incidence of hospital-acquired PI in neonates was 9.8%.^31^

### Older people

An increasingly frequent scenario is the institutionalization of older people. Due to the natural characteristics of the aging process and the clinical issues that limit mobility, PI is considered a concern for the safety of elderly individuals living in these institutions.^32^

A systematic review with meta-analysis showed that the prevalence, incidence, and occurrence of PI acquired in elderly care homes were 8.5%, 11.6% and 14.3%, respectively. The most common stages were Stages 1 and 2. The calcaneal and sacral regions were the most frequently affected sites. Additionally, it was observed that the amount of PI among the older people in these institutions is like that found in hospitalized patients.^32^

### COVID-19

In patients with COVID-19, particularly those during the pandemic admitted to the ICU, the rates of PIs were significantly high. A study in Brazil demonstrated that out of 80 patients admitted to the ICU due to COVID-19, 44 (55%) developed PI during hospitalization.^33^ Another Brazilian study, among the 393 medical records analyzed, identified a prevalence of PI at 167 cases (42.5%), with patients having up to four PI.^34^

In terms of the location of the PI, a difference was noted during the pandemic related to the prone position, in which patients remained for 16 to 24 consecutive hours daily. PI frequently occurred on the bony prominences subjected to this position such as frontal, nasal, pectoral, breast and knee.^35^, ^36^ The incidence of PI ranges from 25.7% to 48.5% in the prone position.^37^

A concern about PI related to the use of personal protective equipment by healthcare professionals has also been reported during the pandemic. A systematic review found a high incidence of these injuries, ranging from 30% to 92.8%, predominantly stage 1 PI^38^ and 58.8% in another study of the same stage.^39^ This is supported by a Brazilian study of ICU professionals, where 91.8% had some form of injury/erythema, primarily in the nasal region (74.1%) and the zygomatic region (45.9%).^40^

## Etiopathogenesis

**Figure 1:**
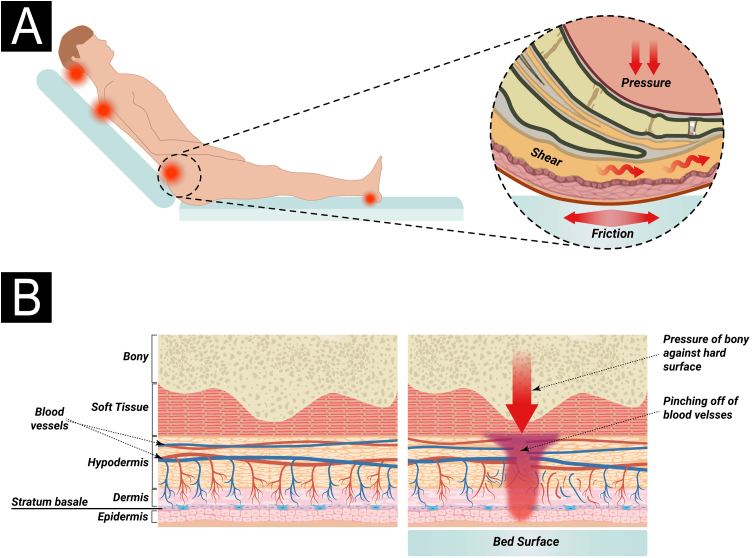
(A) Actions of pressure, friction, and shear over the bony prominence. (B) External pressure and compression of capillaries (from surfaces such as a bed or chair) interfere with local blood flow and, consequently, tissue perfusion, leading to the development of pressure injury. Source: Authors own.

The pathological impact of excessive pressure can be attributed to three factors: the intensity of the pressure, the duration of the pressure, and the capacity of the tissues (including the skin and other structures) to withstand the pressure without sustaining damage.^20^ The influence of capillary pressure and capillary closing pressure is crucial in the development of PIs, as insufficient perfusion due to capillary occlusion can lead to cell and tissue death through ischemia, mechanical deformation, and biochemical stress.^41^ Compression of capillaries by external pressure can interfere with local blood flow and tissue perfusion. This reduces the supply of oxygen and nutrients to the tissues, resulting in the development of PI in areas subjected to mechanical loads composed of prolonged pressure, shear, and friction forces^41^ (Fig. 1A).

In individuals with normal sensitivity, mobility, and mental state, prolonged pressure triggers a feedback response that results in frequent adjustments in body positioning. However, when this feedback response is absent or compromised, sustained and continuous pressure can lead to ischemia, injury, and tissue damage.^42^

Tissue damage occurs due to intense and/or prolonged exposure of soft tissues to sustained mechanical loads, such as compression, traction, or shear forces, or a combination of these loading modes. Sustained loading (quasi-static loading) refers to loads applied continuously over long periods, ranging from minutes to hours or even days^43^ (Fig. 1B).

**Figure 2:**
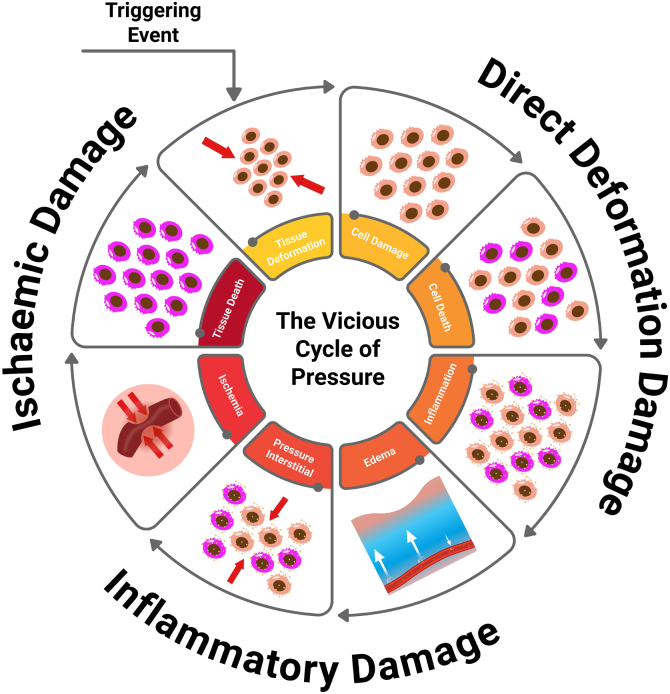
Vicious cycle of PI: direct deformation damage (cellular damage, cell death, and inflammation), inflammatory damage (edema, increased interstitial pressure), ischemic damage (ischemia, tissue death), leading to cellular deformation.

A new conceptual model on the etiopathogenesis of PI was updated by the NPIAP authors in 2022, represented by the PI vicious circle^44^ (Fig. 2).

Figure adapted from Gefen.^44^

The injury mechanism affects various tissues, from deformation to inflammatory cell damage.^45^ Inflammation may intensify due to a pre-existing ischemic condition, especially if reperfusion occurs, for example, immediately after repositioning.^44^, ^46^ Reperfusion after a long period of ischemia can further worsen tissue damage, as it leads to the release of harmful free radicals.^47^ Persistent deformations in cells, blood vessels, and tissues drive this entire damage pathway, impacting the integrity and function of cellular organelles and leading to tissue and organ malnutrition.^43^ These deformations not only cause direct damage to cellular structures but also trigger inflammation and edema, distort the capillary network, reduce nutrient supply to tissues, and obstruct the lymphatic system, impairing the removal of metabolic waste products.^44^ Localized tissue deformations can quickly lead to microscopic damage; however, it may take several hours of continuous pressure for this initial cellular and tissue damage to become clinically visible.^41^

Although pressure is the primary triggering factor, the capacity of soft tissues to tolerate sustained deformations varies depending on the type of tissue.^43^ It is influenced by other factors, known as intrinsic and extrinsic factors, which will be described next.

## Risk factors

The PI occurs due to well-established factors such as pressure, friction, and shear, but several risk factors also contribute to the development of these injuries.^48^ These risk factors are divided into two main domains: extrinsic and intrinsic factors.^49^

Extrinsic factors refer to external conditions or aspects of healthcare practices that can increase the risk of developing PI, such as prolonged pressure, friction and/or shear, moisture, improper handling, medications, hygiene care, and the use of medical devices.^1^, ^50^

Intrinsic factors are related to the individual and physiological characteristics of the patient that can increase their vulnerability to developing PI, such as advanced age, clinical conditions, reduced mobility, urinary and fecal incontinence, hemodynamic status, arterial insufficiency, and malnutrition.^1^

A systematic review with meta-analysis of 39 studies, including over 2.5 million patients worldwide, identified the elderly as a vulnerable patient group. The increase in age is one of the primary risk factors for developing PI, as older individuals tend to have poor skin conditions, inadequate nutrition, and reduced mobility.^21^

Urinary and fecal incontinence results in excess moisture, leading to skin maceration and making it more susceptible to injuries from friction and repositioning.^21^, ^51^

A comprehensive review published in 2024 analyzed several meta-analyses, which systematically highlighted an increased risk of developing PI in patients with diabetes mellitus. Other factors, such as the duration of surgery and the length of stay in the ICU, were also identified as crucial risk elements.^52^

Populations potentially characterized by multiple risk factors include: those who are critically ill and/or in intensive care, individuals with hip fractures, spinal cord injuries, chronic neurological conditions, diabetes mellitus, older people, those residing in long-term care facilities or community care settings, and individuals undergoing trauma and/or prolonged surgery.^1^

## Clinical and laboratory assessment and documentation

When collecting the history of the current PI, it is important to pay attention to the qualitative information about the patient, including location, duration, potential causes or triggering factors, dimensions and changes in size, pain (type, severity, frequency, factors that relieve or worsen), characteristics such as exudate, types of tissue and odor, as well as a history of other PI and scars.^42^

**Figure 3:**
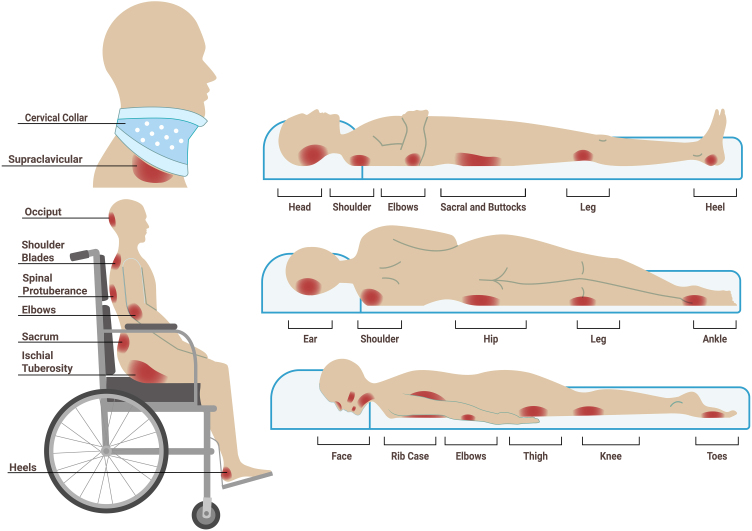
Main bony prominences at risk of developing pressure injuries according to dorsal, lateral, supine, and sitting positions, as well as those related to invasive devices. Sources: Authors' own.

It is necessary to evaluate all bony prominences and critical areas related to positioning and invasive devices (Fig. 3). The main sites affected in adults are typically the sacro-coccigeal region and the heels, the latter mainly from being in the dorsal or seated position.^1^, ^21^, ^53^ Other frequently affected areas include the ischial region in patients who remain seated and the hip (particularly the trochanter) in patients who lie in the lateral position.^1^

In children, the most affected area, especially in infants who are bedridden in the supine position, is the occipital region, followed by the sacral area and heels. Other locations in children include the ears and nose due to the use of medical devices.^1^, ^54^

### Staging

The change in terminology from “pressure ulcer” to “pressure injury” encompasses both intact skin and ulcerated lesions.^17^ Thus, PI describes Stage 1 lesions and deep tissue injuries without ulcerated skin. The staging and examples of anatomical involvement are described in Table 1.^1^

**Table 1:** Classification and definition of pressure injuries according to the European Pressure Ulcer Advisory Panel.^1^

Staging	Definition
Stage 1	Intact skin with non-blanchable erythema, does not lighten with pressure in a localized area. Darkly pigmented skin may not show visible blanching; its color may differ from the surrounding areas. The area may be painful, firm, soft, warmer, or cooler compared to the adjacent tissue (Fig. 4A‒B).
Stage 2	Partial-thickness loss of dermal thickness, appearing as a superficial ulcer with a red-pink wound bed, without necrotic tissue or bruising. It may also present as an intact or ruptured blister. These injuries do not have necrotic tissue and heal by epithelialization rather than granulation tissue. This stage does not apply to moisture-associated skin damage, such as incontinence-associated dermatitis, intertriginous dermatitis, injury related to medical adhesives, or traumatic wounds like skin tears, burns, and abrasions (Fig. 5A‒B).
Stage 3	Full-thickness loss of skin. Subcutaneous tissue may be visible, but bones, tendons, or muscles are not exposed. There may be eschar or slough. Tunneling, undermining, erosions, and epíbole may also be present in Fig. 6A‒B.
Stage 4	Full-thickness tissue loss where exposure may reach bone, tendon, or muscle. Slough or eschar may be present in some parts of the wound bed. Often includes erosion and tunneling. Exposed bone/tendon is visible or directly palpable, which may lead to osteomyelitis (Fig. 7A‒B).
Deep Tissue Pressure Injury	Persistent discoloration of intact skin or the presence of a blister (bulla), characterized by a translucent lesion that may contain blood-stained, clear, serous, or purulent fluid, suggesting possible deep damage to the underlying tissue (Fig. 8A‒B).
Unstageable Pressure Injury	Full-thickness skin and tissue loss in which the base of the wound is covered by eschar (yellow, beige, gray, green, or brown) and/or necrotic tissue (beige, brown, or black) on the wound bed. The stage cannot be determined until the necrotic tissue or eschar is removed to reveal the base of the wound. Excludes PI reclassified to Stage 3 or 4 after exposure or debridement (Fig. 9A‒B).
Medical Device-Related Pressure Injury	Caused by using devices designed and utilized for diagnostic or therapeutic purposes. These injuries exhibit a pattern or shape consistent with the device that applies pressure to the skin. They should be classified using a recognized staging system, similar to other PI (Fig. 10A).
Pressure Injury to Mucous Membranes	These injuries occur on the moist membranes lining the respiratory, gastrointestinal, and genitourinary tracts. Medical devices, such as tubes and stabilization equipment primarily cause them. Due to the unique anatomy of these tissues, these injuries cannot be classified into Stage 1 through Stage 4 (Fig. 10B).

PI is not limited to the skin; it can also occur on mucous membranes, including the respiratory, gastrointestinal, and genitourinary tracts. Mucosal PI is primarily associated with medical devices, typically caused by tubes and/or their stabilization equipment exerting sustained compressive and shear forces on vulnerable mucous membranes and underlying tissues.^1^

**Figure 4:**
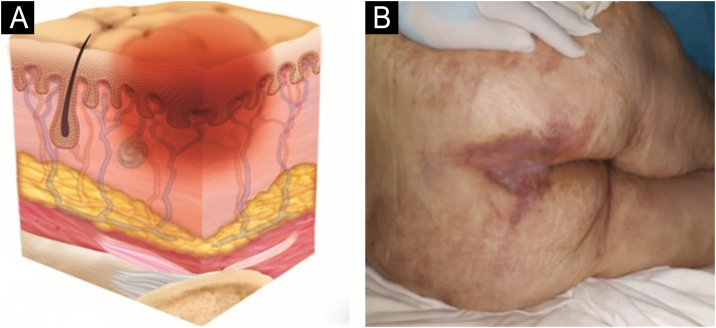
(A) Stage 1 pressure injury. Source with permission of the National Pressure Injury Advisory Panel. (B) Patient with non-blanchable erythema in the sacral region. Source: Authors' own.

**Figure 5:**
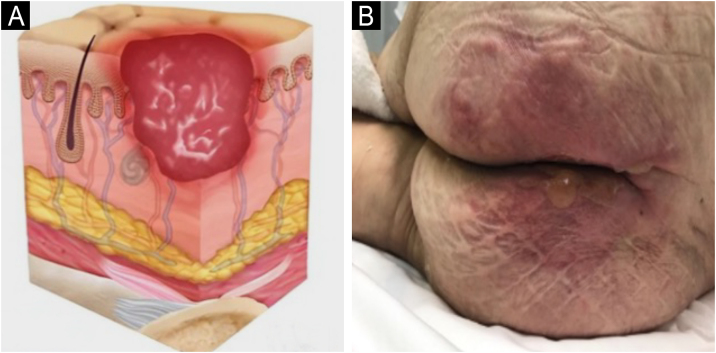
(A) Stage 2 pressure injury. Source with permission of the National Pressure Injury Advisory Panel. (B) Patient with partial-thickness skin loss and an intact blister in the gluteal region. Source: Authors' own.

**Figure 6:**
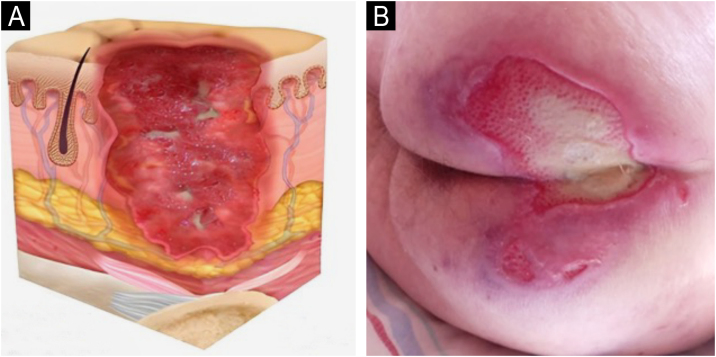
(A) Stage 3 pressure injury. Source with permission of the National Pressure Injury Advisory Panel. (B) Patient with full-thickness dermal loss in the gluteal region and the presence of slough. Source: Authors' own.

**Figure 7:**
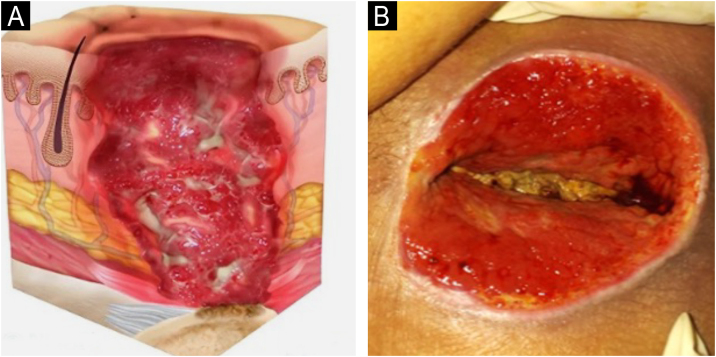
(A) Stage 4 pressure injury. Source with permission of the National Pressure Injury Advisory Panel. (B) Patient with full-thickness skin loss in the sacral region and exposure bone. Source: Authors' own.

**Figure 8:**
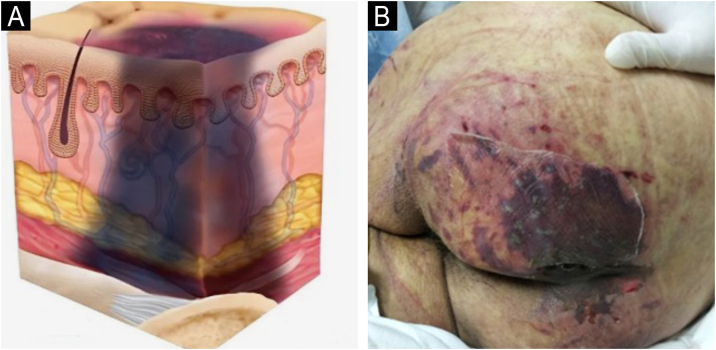
(A) Deep tissue pressure injury. Source with permission of the National Pressure Injury Advisory Panel. (B) Patient with extensive purpuric lesion in the gluteal region. Source: Authors' own.

**Figure 9:**
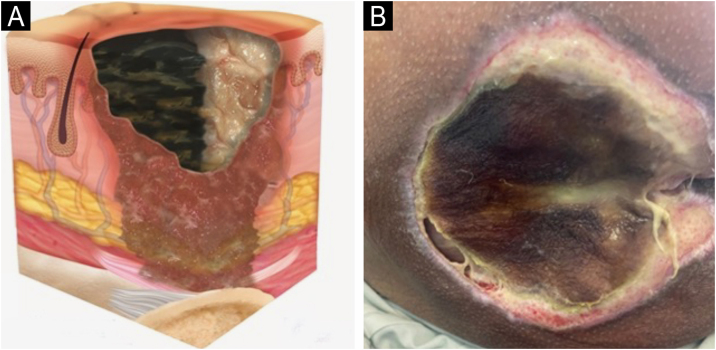
(A) Unstageable pressure injury. Source with permission of the National Pressure Injury Advisory Panel. (B) Patient with liquefactive necrosis and an area of coagulative necrosis in the sacral region, making it impossible to assess the depth of the pressure injury. Source: Authors' own.

**Figure 10:**
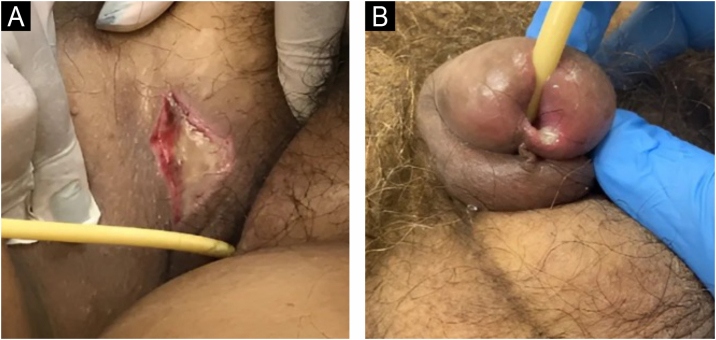
(A) Medical device-related pressure injury in a patient with a pressure injury on the inner surface of the right thigh caused by pressure from an indwelling urinary catheter. (B) Urethral mucosal pressure injury in a patient with an indwelling urinary catheter. Source: Authors' own.

The representation of the affected skin layers and images of representative pressure injuries according to each classification are in Figure 4, Figure 5, Figure 6, Figure 7, Figure 8, Figure 9, Figure 10.

Evidence-based recommendations for additional assessment techniques, such as skin temperature evaluation, subepidermal moisture measurement, and skin tone color charts, should be used when assessing skin and tissues and classifying PI, particularly in darker skin tones.^1^ Assessment of the skin using subepidermal moisture measurement scanners has gained international attention as it can detect the onset of PI approximately five days before becomes visually apparent,^55^ making it a promising advancement in the clinical practice of PI prevention.^56^

Some tools guide the assessment and documentation of PIs, such as the widely used PUSH (Pressure Ulcer Scale for Healing), which provides a guide for evaluating exudate, size, and tissue type, serving as a support for clinical progression.^57^, ^58^ Another more current and comprehensive tool that supports professionals is the Bates-Jensen Wound Assessment Tool (BWAT)^59^ – adapted for Brazil in 2015^60^ and its reliability was internationally validated in 2019.^61^ The BWAT consists of 13 wound characteristics: size, visible depth, wound edges, undermining and tunneling, type and amount of necrotic tissue, type and amount of exudate, surrounding skin discoloration, peripheral tissue edema, peripheral tissue induration, granulation tissue, and epithelialization.^60^, ^61^

### Laboratory aspects

Laboratory tests help assess conditions that can hinder or delay PI healing, such as infection, anemia, poor nutritional status, and diabetes mellitus. It is recommended that a comprehensive metabolic panel, a complete blood count with differential, albumin and prealbumin (to evaluate nutritional status), and hemoglobin A1C be performed.^62^

Although swabs to assess for infection remain the most common method for wound sample collection due to their simplicity and low cost,^63^ wound cultures from superficial swabs may not be clinically valuable. They often reflect colonization rather than infection, leading to false-positive results.^64^, ^65^, ^66^

Tissue biopsy is considered the gold standard due to its precision, although it is more invasive, costly, and requires specialized skills for its performance.^63^ Additionally, biopsy has greater sensitivity for wounds infected with multidrug-resistant bacteria.^65^

## Differential diagnoses

### Incontinence-associated dermatitis

Lesions caused by excessive moisture (sweat, exudate, and bodily fluids) compromise the epidermal barrier and can cause skin damage that may be confused with PI.^1^ Incontinence-Associated Dermatitis (IAD) is an irritant contact dermatitis and is the most common form of moisture-associated skin damage, as chronic exposure of the skin to urine and feces chemically injures the skin.^1^

**Figure 11:**
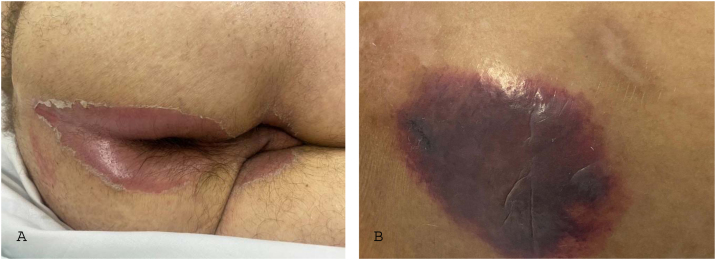
Differential diagnosis of pressure injury. (A) Incontinence-associated dermatitis in a patient with urinary and fecal incontinence who developed intense erythema in the regions intergluteal, perianal and scrotum. (B) Kennedy’s terminal ulcer in a patient with multiple organ failure who rapidly developed (within 24 hours) a purpuric plaque in the sacral region.

Source: Authors' own.

The IAD is characterized as an inflammatory dermatosis with erythema, exudation, and excoriation.^67^ It also presents with persistent pigmentation changes, edema, maceration, vesicles, or blisters.^68^ It affects the perineum, the lower folds of the buttocks, the scrotal region, the thighs, and sometimes even the abdomen (Fig. 11A).

### Kennedy terminal ulcer

End-of-life injuries are those that appear hours or weeks before a patient's death.^69^ Many synonyms describe the same type of injury, with the most well-known term being the eponym “Kennedy Terminal Ulcer” (KTU). KTU was first reported by Karen Lou Kennedy^70^ describing a discolored skin patch (purple, red, blue or black) usually located in the sacral or coccyx region, although it can affect other body parts, with a butterfly, pear, or horseshoe shape (Fig. 11B). Its progression is very rapid, with full-thickness ulcers evolving over the course of hours or a few days, indicating the imminence of death.^1^

There is debate as to whether KTU is a particular type of PI. However, there is a difference in the pathophysiology between them.^71^ PI is caused by unrelieved pressure, often accompanied by shear and friction, resulting in local ischemia and tissue damage. On the other hand, KTU is caused by hypoperfusion and multiorgan failure, and the hypoperfusion of the skin results in visible signs of tissue death. In this scenario, the ischemia associated with multiorgan failure, rather than the damaging effects of unrelieved pressure or shear, results in an unavoidable skin ulcer.^69^

Due to similar purple or brown discoloration and location, it can be challenging to differentiate it from deep tissue PI.^72^

### Final considerations

This article aimed to support healthcare professionals and dermatologists in identifying risk factors and mechanisms of PI based on the most current concepts available in the scientific literature. The primary focus was providing resources to help them holistically assess, prescribe, and monitor patients. Additionally, it aimed to assist in evaluating each risk classification and distinguishing other skin conditions like PI.

Some key points of this review include: 1) The prevalence of PI remains high in the global population, with emphasis on patients in ICUs, institutionalized older adults, individuals with hip fractures, trauma or prolonged surgeries, spinal cord injuries, and chronic neurological conditions; 2) Children and neonates are also at risk of PI; 3) PI is not limited to the skin; it can also occur on mucous membranes primarily associated with medical devices; 4) Temperature assessment, subepidermal moisture measurement, and skin tone color charts can be used when assessing skin and tissue and grading PI, particularly in darker skin tones; 5) In cases of PI with suspected infection, wound cultures from superficial swabs are not indicated. A biopsy for microbiological analysis has greater sensitivity in detecting wounds infected with multidrug-resistant bacteria.

The authors invite you to read the subsequent article, which addresses the main complications inherent to PI, practical preventive measures, conventional treatments, and advanced technologies currently in use. Additionally, the next article provides a practical guide focusing on combating biofilm and updating debridement techniques.

## Research data availability

The entire dataset supporting the results of this study was published in this article.

## Scientific associate editor

Hiram Larangeira de Almeida Jr.

## Financial support

None declared.

## Authors' contributions

Bruna Cristina Velozo: Study design and planning; literature review; article writing; critical literature review; critical manuscript review; approval of the final version of the manuscript.

Michelle Venâncio Hong: Study design and planning; literature review; article writing; critical literature review; critical manuscript review; approval of the final version of the manuscript.

Larissa Cassiano Bernardo: Study design and planning; literature review; article writing; critical literature review; critical manuscript review; approval of the final version of the manuscript.

Meire Cristina Novelli e Castro: Study design and planning; literature review; article writing; critical literature review; critical manuscript review; approval of the final version of the manuscript.

Luciana Patrícia Fernandes Abbade: Study design and planning; literature review; article writing; critical literature review; critical manuscript review; approval of the final version of the manuscript.

## Conflicts of interest

None declared.
